# Identification and Analysis of Chemical Constituents and Rat Serum Metabolites in* Lycopodium clavatum* Using UPLC-Q-TOF/MS Combined with Multiple Data-Processing Approaches

**DOI:** 10.1155/2019/5165029

**Published:** 2019-07-02

**Authors:** Xin Li, Mingqin Kang, Ningning Ma, Tan Pang, Yanjun Zhang, Hua Jin, Zhen Yang, Lili Song

**Affiliations:** ^1^School of Chinese Materia Medica, Tianjin University of Traditional Chinese Medicine, Jian Kang Chan Ye Yuan, Jinghai Dist., Tianjin 301617, China; ^2^Changchun Customs (Former Jilin Inspection and Quarantine Bureau), Changchun 130062, China; ^3^Pharmaron (Beijing) Inc., Beijing 100176, China; ^4^Tianjin State Key Laboratory of Modern Chinese Medicine, Tianjin Key Laboratory of Traditional Chinese Medicine Pharmacology, Tianjin Key Laboratory of TCM Chemistry and Analysis, Tianjin University of Traditional Chinese Medicine, Jian Kang Chan Ye Yuan, Jinghai Dist., Tianjin 301617, China; ^5^College of Traditional Chinese Medicine, Tianjin University of Traditional Chinese Medicine, Jian Kang Chan Ye Yuan, Jinghai Dist., Tianjin 301617, China

## Abstract

*Lycopodium clavatum* is a dry whole grass of* Lycopodium japonicum* Thunb.; it has been extensively used to anti-inflammatory, antioxidant, and antimicrobial actions and inhibits acetylcholinesterase activity. However, it lacks further compounds research of* Lycopodium clavatum* in* vivo* and in* vitro*. In this work, a rapid method was established using the ultra high performance liquid chromatography with quadrupole-time-of-flight mass spectrometry (UPLC-Q-TOF/MS) combined with multiple data-processing approaches for compounds analysis of* Lycopodium clavatum* in* vitro* and in* vivo*. Finally, 30 peaks were characterized in 75% ethanol extract of* Lycopodium clavatum* and 17 peaks were characterized in rat plasma that including 12 prototype compounds and 5 metabolites. Methylation and demethylation are the main transformation reactions of* Lycopodium clavatum* in rat serum. This work could be helpful for understanding the complex compounds of* Lycopodium clavatum* and further analyzing the pharmacological studies of active compounds.

## 1. Introduction

*Lycopodium clavatum *(LC) is a dry whole grass of* Lycopodium japonicum *Thunb.; it has been widely used to anti-inflammatory [1], antioxidant, and antimicrobial actions [2] and inhibits acetylcholinesterase activity [3–5], meanwhile, it has antioxidative, antiproliferative, and biochemical effects for HepG2 cells [6]. In recent years, literature reports of LC pay more attention to analyzing component in* vitro*. LC mainly contains alkaloids [7] and triterpenoids [8–11]. Alkaloids are divided into four types with the structural characteristics, including lycopodine, lycodine, fawcettimine, and miscellaneous. Four types of mother nucleus all have the same configuration, so it is inferred that there is a common biological origin and transformation relationship between them [12, 13]. So far, there have been few reports on the chemical constituents of LC, and there is almost no research on the blood components in the body. Therefore, it is especially important for the chemical composition of LC in* vivo* and in* vitro*. At present, LC-MS is often used to analyze and determine the component of traditional Chinese medicine, especially the technology of UPLC-Q-TOF/MS [14, 15]. UPLC-Q-TOF/MS combined with multiple data processing approaches can improve efficiency and obtain abundant information, which can be utilized as a practical model for the study of component [16, 17]. The combination of the technology has the advantages of high efficiency, wide molecular mass range, high sensitivity, low error rate, and high reproducibility [18, 19]. In this work, a rapid method will be established for the compounds analysis of LC in* vitro* and in* vivo* by the ultra high performance liquid chromatography (UPLC) with quadrupole-time-of-flight mass spectrometry combined with multiple data-processing approaches, which can be used to further analyze the chemical composition in* vivo* and in* vitro*.

## 2. Methods and Materials

### 2.1. Chemicals and Materials

*Lycopodium clavatum* was purchased from Beijing Huamiao Pharmaceutical Co. Ltd. It was identified as a dry whole grass of* Lycopodium japonicum *Thunb. by Professor Tianxiang Li from the College of Traditional Chinese Medicine of Tianjin University of Traditional Chinese Medicine. Methanol and acetonitrile (HPLC grade) were obtained from Sigma-Aldrich (America). Formic acid (HPLC grade) was purchased from Aladdin (America). Water was acquired from Wahaha (China). Ethanol (analysis grade) was purchased from Beijing Chemical Works (China). Male SD rats (200 ± 10g) were obtained from Beijing Huakangkang Biotechnology Co. Ltd.

### 2.2. Preparation of Lycopodium clavatum Samples In Vitro

*Lycopodium clavatum* were added 10 times and 6 times 75% (*v/v*) ethanol, respectively, and were reflux extracted twice for 1 h each time. After being extracted, the extract was filtered and condensed to 1 g/mL. The concentrate was diluted to 10mg/mL by water with acetonitrile: 0.1% formic acid=1:1 (*v/v*) and through 0.22* μ*m filter membranes before analysis.

### 2.3. Animal Experiments

Male SD rats were acclimatized for 1 week before the experiment and collected blank blood. The suspension solution of* Lycopodium clavatum* was intragastrical administered at a dose of 10.8 g/kg for 3 days. After the last administration, the rats were collected blood by orbital vein at 15 min, 1 h, 4 h, and 8 h. All samples were centrifuged at 3,000 rpm for 15 min at 4°C and the plasma supernatant was pipetted.

### 2.4. Preparation of Plasma Samples In Vivo

For analysis, plasma was consolidated including the different blood collection points and 500* μ*L plasma was added 1 mL acetonitrile for protein precipitation. After vortexed vigorously for 1 min, the samples were centrifuged at 13, 000 rpm for 15 min at 4°C and pipetted plasma supernatant. 1 mL acetonitrile was added to the residue and the above operation was repeated one time. Two-part supernatants were combined and was evaporated to dried under N_2_ at 45°C. The residue was dissolved with 100* μ*L methanol, vortex-mixed for 1 min before being centrifuged at 13,000 rpm at 4°C for 15 min and then pipetted plasma supernatant for analysis.

### 2.5. Chromatographic Analysis

The supernatant of sample was using A Waters ACQUITY I Class UPLC coupled to a Waters Xevo G2 Q TOF mass spectrometer, where the parameters were as follows. The chromatographic column was ACQUITY UPLC HSS T3 Column (2.1×100 mm, 1.8* μ*m, Waters Corporation, America). Mobile phases consisted of acetonitrile with 0.1% formic acid (phase A) and ultrapure water with 0.1% formic acid (phase B). Whole analysis process, runtime was 25min, the flow rate was set at 0.2 mL/min, and the temperature of the column was set at 40°C. The linear elution gradient program was used as follows: 0.5-1.5 min, 99–90% B; 1.5-4 min, 90-85% B; 4-8 min, 85-70% B; 8-9 min, 70-50% B; 9-11.5min, 50-20% B; 11.5-14 min, 20-0% B; 14-17 min, 0%B; 17-18 min, 0-99%B. The injection volume was set at 5*μ*L.

### 2.6. MS Conditions

An UPLC-Q-TOF/MS (waters, America) instrument is equipped with both positive and negative ESI source for sample analysis. The samples were measured from 50 to 1,000 mass-to-charge ratios (*m/z*) and the temperature of nitrogen was set to 350°C. The temperature of dissolvent was set to 80°C and the gas flow was set to 600 L/h. The capillary voltage was set to 3.0kV in positive ion mode, and it was set to 2.5 kV in negative ion mode.

### 2.7. Data Processing

A rapid analysis of the extract of* Lycopodium clavatum* was carried out to obtain the retention time and the exact molecular weight of each component. The possible elemental composition was obtained through the MassLynx V4.1 workstation, and only those formulas with an error less than 10 ppm were accepted. The probable molecular formulas were matched with the relevant literature and databases such as SciFinder and MassBank. Meanwhile, to combine with MS and MS/MS information inferred the compound and its structure.

Raw spectrometric data were processed by MassLynx software (version 4.1, Waters Co., Cicero, America) and converted to a data matrix contain sample name, t-*m/z* and peak intensity. Data was further processed, including peak matching, peak alignment, and normalization, before multivariate analysis. Multivariate analysis was performed using a combination of principal component analysis (PCA) and the orthogonal projection to least-squared discriminant analysis (OPLS-DA) by SIMCA-P software (version 12.0), and then was further performed using* t*-test for Microsoft Excel software (version 2010). Metabolites were retained in the model based on a combination of variable importance in projection (VIP) greater than 1.0 and P value of less than 0.05.

MetaboLynx XS version 4.1 (Waters, Milford, America) can be used to find the unknown drug metabolites in* vivo*. Biotransformation reactions were set according to its different structure and the peak intensity threshold of 40 with tolerance was set at 0.05 Da. The result of possible metabolites and its biotransformation reactions were determined by the relevant literature and MS and MS/MS information.

## 3. Results and Discussion

### 3.1. Establishment of Compounds Database

To quickly and easily identify the compounds of* Lycopodium clavatum*, we established compounds database by collecting information of literature in ten years [7–11, 20–24] and searching web databases including Pubmed, SciFinder, and MassBank. The database includes 121 prototype compounds of* Lycopodium clavatum *that was shown in Table S1, including name, molecular formula, molecular weight, and structure, to support the results of identification for further analysis in* vitro* and in* vivo*.

### 3.2. UHPLC–MS Analysis and Identification of Lycopodium clavatum In Vitro

**Figure 1:**
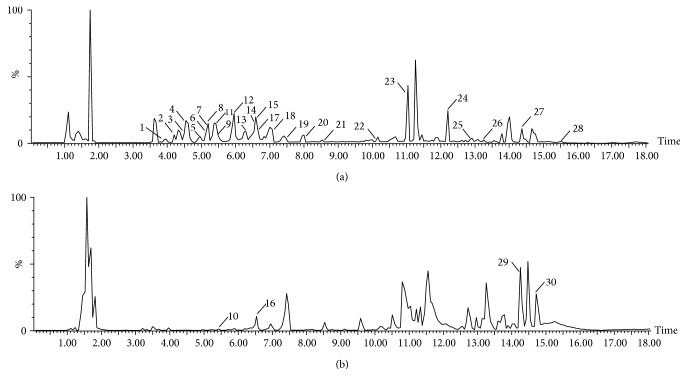
ESI base peak ion (BPI) chromatogram of the extract sample of* Lycopodium clavatum* extracts analyzed by UPLC–Q-TOF/MS in both positive (a) and negative (b) ion mode.

Under the above MS and chromatographic conditions, the chromatogram of the base peak ion (BPI), the extract sample of* Lycopodium clavatum* was shown in Figure 1. According to the chromatogram, the baseline was stable and the chromatographic peaks were well separated, indicating that the various components of* Lycopodium clavatum* extract could be well separated in the data collection range.

**Figure 2:**
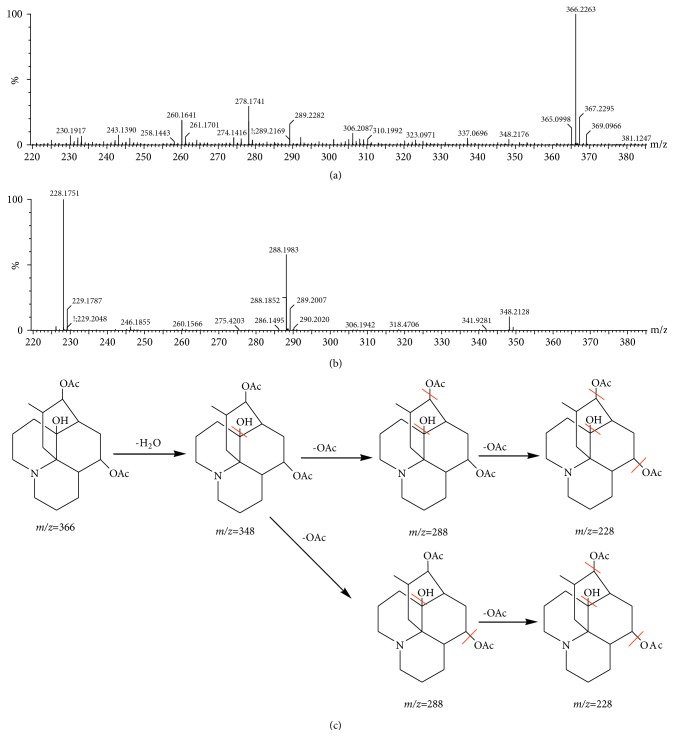
The mass spectra of acetyllycofawcine analyzed by UHPLC–Q-TOF-MS. the MS spectrum (a); the MSMS spectrum (b); proposed fragmentation pathways of acetyllycofawcine (c).

The potential compounds were identified by comparing the information of MS and MS/MS; meanwhile, the result was associated with the relevant literature and databases for further determination. For Examples, the precise molecular weight was 366.2274 and the retention time was 7.89 min. The main fragment ions that were analyzed by the MS/MS screening were observed at* m/z* 348[M+H-H_2_O] ^+^, 288[M+H -C_2_O_2_H_4_] ^+^, 228[M+H-C_4_O_4_H_8_]^+^ in the positive ion spectrum. Based on MS and MS/MS data obtained and the analysis of elemental composition, the molecular formula of the ion was speculated of C_20_H_31_NO_5_. Combining with literature [24] it was inferred from acetyllycofawcine. Mass spectrum and proposed fragmentation pathway of acetyllycofawcine were shown in Figure 2. Finally, a total of 30 compounds were identified (characteristic peaks were labeled on the BPI chromatographic), including 21 alkaloids, 7 triterpenoids, and 2 other classes compound. The detailed information of compound name, molecular weight, molecular formula, and characteristic peak was shown in Table 1.

### 3.3. Multivariate Statistical Analysis and Identification of Plasma Sample after Oral Lycopodium clavatum In Vivo

**Figure 3:**
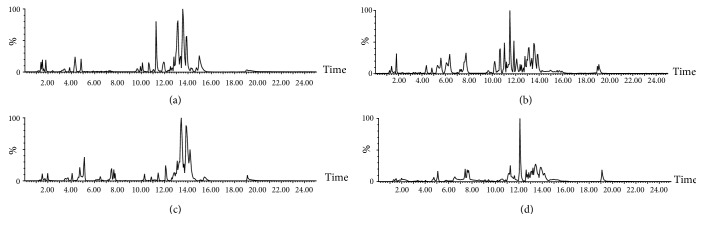
ESI base peak ion (BPI) chromatogram of the plasma sample of* Lycopodium clavatum* extracts analyzed by UPLC–Q-TOF/MS in both positive (a) and negative (b) ion mode; ESI base peak ion (BPI) chromatogram of the control plasma sample of* Lycopodium clavatum* extracts analyzed by UPLC–Q-TOF/MS in both positive (c) and negative (d) ion mode.

In order to analyze the compounds of* Lycopodium clavatum* in plasma, the two groups were compared between the plasma sample of* Lycopodium clavatum* group and the control group that the chromatogram of BPI was shown in Figure 3, and then multivariate statistical analysis was used to further discover the prototype compounds in* vivo*.

**Figure 4:**
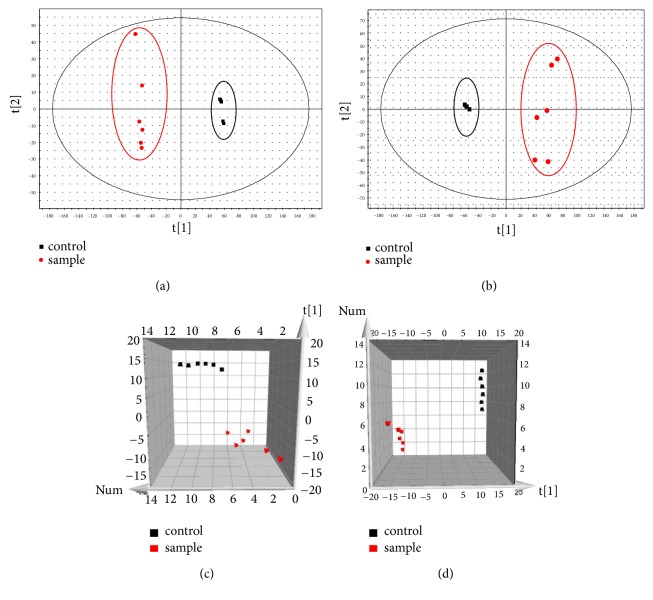
Multivariate statistical analysis and identification of plasma sample of* Lycopodium clavatum*. PCA of* Lycopodium clavatum* group vs. control group in positive mode (a) and negative mode (b); 3D-OPLS-DA of* Lycopodium clavatum* group vs. control group in positive mode (c) and negative mode (d).

PCA is a chemometric method that uses the idea of dimensionality reduction to transform multiple indicators into several comprehensive indicators with little information loss. In the PCA score (Figure 4), it could be observed that the plasma samples of drug-administered group and blank group were divided into two discrete groups, indicating that there was a significant difference in the drug-administered group and blank group. In order to further analyze the metabolic differences between the blank group and the drug-administered group in plasma samples, an in-depth analysis of the data (OPLS-DA) is required. According to the OPLS-DA score (Figure 4), it was clearly observed that the two groups plasma samples were divided into 2 discrete groups, which was better than the score of PCA. VIP value reflects the contribution of the variable in the classification. The larger the VIP value, the greater the contribution of the variable to the entire model classification. Generally, VIP value is greater than 1 as the characteristic variable of the metabolic. The value of VIP greater than 1 was screened and further analyzed. Finally, a total of 12 compounds were identified by the data of MS, MS/MS information, and the relevant literature and databases. They were lycojaponicumin C (6), huperzine E (8), des-N-methyl-*α*-obscurine (10), *α*-obscurine (11), lycopodine (13), 8*β*-acetoxy-12*β*-hydroxy-lycopodine or 8*β*-acetoxy-11*α*-hydroxy-lycopodine or 8*β*-hydroxy-11*α*- Acetoxylycopodine (14), lycodoline (16), *α*-lofoline or fawcettiine (17), acetylfawcettiine (20), japonicumin B or lycoclavanin (23), 3*R*, 21*α*, 24-trihydroxyserrat-14-en-16-one (24) and lycojaponicuminol C (26). Among them, *α*-obscurine (11) and lycodoline (16) were identified by comparison with the information of the standard, including retention time, MS and MSMS data. Next, the more standard will be purchased and used to further qualitative analysis of the compounds of* Lycopodium clavatum*.

### 3.4. Metabolites Identified Using MetaboLynx Tool

After the drug gets into the body, some components exist as the form of prototype, but most of them are structurally modified based on the original drug components, such as methylation and glutathione conjugation. The metabolite is structurally similar to the prototype drug and has characteristic mass spectrometry fragmentation of the prototype drug, so the structure of the metabolite can be inferred by characteristic fragment between the prototype and metabolite. Using MetaboLynx XS to process the data to analyze the plasma samples, most of the potential metabolites were identified. Finally, 5 metabolites were detected from the* Lycopodium clavatum.* The molecular weight of lycojaponicumin C (6) was 274.1811 and the formula was C_17_H_23_NO_2_. The precise molecular weight of M1 was 260.1653 that had a neutral loss of 14 Da compared with lycojaponicumin C (6) and the main fragment ions was 167, 158, and 125 that were the same as the main fragment ions of lycojaponicumin C (6); it is inferred that M1 is a metabolite of the demethylation of the lycojaponicumin C. Finally, a total of 5 metabolites were identified the same way. M2 is a metabolite of the methylation of the palhinine A, M3 is a metabolite of the methylation of the (15*R*)-14,15-dihydroepilobscurinol, M4 is a metabolite of the methylation of lycodoline, and M5 is a metabolite of the demethylation of *α*-lofoline. The detailed information of prototype, metabolite name, molecular weight, and molecular formula was given in Table 2.

## 4. Conclusion

In this work, we demonstrated a systematic and reliable analytical method using UPLC–Q-TOF-MS coupled with multiple data processing approaches for rapidly screening and identifying the chemical constituents of* Lycopodium clavatum* both in* vitro* and in* vivo*. In* vitro*, 30 peaks were characterized in 75% ethanol extract of* Lycopodium clavatum.* In* vivo*, 17 peaks were characterized in rat plasma that including 12 prototype compounds and 5 metabolites. Methylation and demethylation are the main transformation reactions of* Lycopodium clavatum* in rat plasma. This work could be helpful to understand the complex compounds of* Lycopodium clavatum* and further analyze the pharmacological studies of active compounds.

## Figures and Tables

**Table 1:** Identification of the components from *Lycopodium clavatum* in *vitro*.

NO.	t_R_(min)	Measured Mass (*m*/*z*)	Fragment ions (*m*/*z*)	Formula	Error (ppm)	Identification
1	3.8002	278.1756	260, 162, 134	C_16_H_23_NO_3_	0	8*β*-hydroxylycoposerramine
2	4.1863	266.2119	248, 230, 202	C_16_H_27_NO_2_	0.38	aeacetylfawcettiine
3	4.6209	292.1914	274, 203, 135	C_17_H_25_NO_3_	0.34	palhinine A
4	4.6343	273.1963	243, 200, 186	C_17_H_24_N_2_O	1.46	obscurinene
5	4.9963	296.185	278, 260, 234	C_16_H_25_NO_4_	4.05	4*α*,8*β*,12*β*-trihydroxylycopodine
6	5.1711	274.1811	231, 167, 158	C_17_H_23_NO_2_	1.46	lycojaponicumin C
7	5.2178	262.1807	234, 206, 164	C_16_H_23_NO_2_	0	lycopoclavamine A
8	5.2195	260.1661	246, 24, 216	C_16_H_21_NO_2_	3.84	huperzine E [4]
9	5.3981	280.1912	262, 244, 163	C_16_H_25_NO_3_	0.36	6*α*,8*β*-dihydroxylycopodine or 4*α*,8*β*-dihydroxylycopodine
10	5.5543	261.1966	245, 202, 188	C_16_H_24_N_2_O	0.38	des-N-methyl-*α*-obscurine
11	5.8878	275.2125	244, 188, 148	C_17_H_26_N_2_O	0.73	*α*-obscurine
12	6.0787	278.212	260, 217, 189	C_17_H_27_NO_2_	0	(15*R*)-14,15-dihydroepilobscurinol
13	6.2025	248.2016	230, 145, 105	C_16_H_25_NO	0.81	lycopodine
14	6.4302	322.2037	244, 216, 176	C_18_H_27_NO_4_	5.9	8*β*-acetoxy-12*β*-hydroxy-lycopodine or 8*β*-acetoxy-11*α*-hydroxy-lycopodine or 8*β*-hydroxy-11*α*-acetoxy-lycopodine
15	6.4922	246.1854	218, 176, 124	C_16_H_23_NO	1.62	anhydrolycodoline
16	6.5863	264.1966	228, 242, 191	C_16_H_25_NO_2_	0.76	lycodoline
17	6.7897	308.2226	248, 230, 159	C_18_H_29_NO_3_	0	*α*-lofoline
18	7.3916	276.1962	218, 166, 150	C_17_H_25_NO_2_	0.72	lycoflexine
19	7.8795	366.2274	348, 288, 228	C_20_H_31_NO_5_	3.82	acetyllycofawcine
20	8.2908	350.2324	290, 230	C_20_H_31_NO_4_	2.28	acetylfawcettiine
21	8.5172	195.066	167, 152, 115	C_10_H_10_O_4_	1.54	ferulic acid
22	10.2069	505.3513	487, 263, 199	C_30_H_48_O_6_	3.17	16-oxolyclanitin
23	11.0118	489.3571	471. 453, 345	C_30_H_48_O_5_	1.84	japonicumin B or lycoclavanin
24	12.1919	473.362	102,111,167	C_30_H_48_O_4_	2.32	3*R*,21*α*,24-trihydroxyserrat-14-en- 16-one
25	12.6387	475.377	457, 410, 237	C_30_H_50_O_4_	3.58	Lycoclaninol [4] or japonicumin A
26	13.0973	461.362	443,441,102	C_29_H_48_O_4_	2.38	lycojaponicuminol C
27	14.2839	445.3667	279,427,319	C_29_H_48_O_3_	3.37	26-nor-8-oxo-*α*-onocerin
28	15.3438	457.3664	439,397	C_30_H_48_O_3_	3.93	lycojaponicuminol A
29	15.5437	459.3828	441,398,397	C_30_H_50_O_3_	2.18	3-epilycoclavanol or 3*β*,21*β*,24-3-trihydroxyserrat-14-ene or 3*α*,21*β*,24-4-trihydroxyserrat-14-ene
30	16.1058	603.4045^[M-H]^	116,132,134	C_39_H_56_O_5_	0.66	lycojaponicuminol D

**Table 2:** Identification of the metabolites from *Lycopodium clavatum* in *vivo*.

NO.	t_R_(min)	Measured Mass (*m*/*z*)	Metabolite Name	Formula	Error (ppm)	Prototype
M1	5.2	260.1653	demethylation	C_16_H_21_NO_2_	1	lycojaponicumin C
M2	6.95	306.2077	methylation	C_18_H_27_NO_3_	2.6	palhinine A
M3	9.17	292.2287	methylation	C_18_H_29_NO_2_	3.7	(15*R*)-14,15-dihydroepilobscurinol
M4	7.12	278.2127	methylation	C_17_H_27_NO_2_	2.6	lycodoline
M5	3.57	294.2076	demethylation	C_17_H_27_NO_3_	2.4	*α*-lofoline

## Data Availability

The data used to support the findings of this study are included within the article.

## Supplementary Materials

Supplementary Materials Table S1: The database of 121 prototype compounds of Lycopodium clavatum.
