# Supplementary material for: Identification and Analysis of Chemical Constituents and Rat Serum Metabolites in Lycopodium clavatum Using UPLC-Q-TOF/MS Combined with Multiple Data-Processing Approaches
Source: Evid Based Complement Alternat Med. 2019 Jul 2;2019:5165029. doi: 10.1155/2019/5165029 (PMC6633958; doi:10.1155/2019/5165029)
Supplement: Supplementary Materials — Table S1: The database of 121 prototype compounds of Lycopodium clavatum. [file 5165029.f1.docx]

Table S1 The database of 121 prototype compounds of *Lycopodium clavatum*

| **No.** | **Name** | **Mass (m/z)** | **Formula** | **Structure** | |  |
| --- | --- | --- | --- | --- | --- | --- |
| **alkaloid of lycopodine type** | | | | | |  |
| 1 | anhydrolycodoline [25] | 245.178 | C_16_H_23_NO |  | |  |
| 2 | lycopodine [25] | 247.1936 | C_16_H_25_NO |  | | |
| 3 | huperzine E [21] | 259.1572 | C_16_H_21_NO_2_ |  | |  |
| 4 | lycoposerramine M [22] | 263.1885 | C_16_H_25_NO_2_ |  | |  |
| 5 | clavolonine [22] | 263.1885 | C_16_H_25_NO_2_ |  | |  |
| 6 | lycodoline [20] | 263.1885 | C_16_H_25_NO_2_ |  | |  |
| 7 | lucidioline [20] | 263.1885 | C_16_H_25_NO_2_ |  | |  |
| 8 | daeacetylfawcettiine [25] | 265.2042 | C_16_H_27_NO_2_ |  | |  |
| 9 | 8β-hydroxyhuperzine E [25] | 275.1521 | C_16_H_21_NO_3_ |  | |  |
| 10 | 8β-hydroxylycoposerramine [25] | 277.1678 | C_16_H_23_NO_3_ |  | |  |
| 11 | 6α,8β-dihydroxylycopodine [25] | 279.1834 | C_16_H_25_NO_3_ |  | |  |
| 12 | 4α,8β-dihydroxylycopodine [25] | 279.1834 | C_16_H_25_NO_3_ |  | |  |
| 13 | 8β-hydroxylycodoline [25] | 279.1834 | C_16_H_25_NO_3_ |  | |  |
| 14 | 11β-hydeoxy-12-epilycodoline [25] | 279.1834 | C_16_H_25_NO_3_ |  | |  |
| 15 | lycoposerramine G [25] | 279.1834 | C_16_H_25_NO_3_ |  | |  |
| 16 | miyoshianine A [20] | 279.1834 | C_16_H_25_NO_3_ |  | |  |
| 17 | 4α,8β,12β-trihydroxylycopodine [25] | 295.1784 | C_16_H_25_NO_4_ |  | |  |
| 18 | miyoshianine C [20] | 295.1784 | C_16_H_25_NO_4_ |  | |  |
| 19 | α-lofoline [22] | 305.1991 | C_18_H_27_NO_3_ |  | |  |
| 20 | 8β-acetoxy-12β-hydroxy-lycopodine [22] | 321.194 | C_18_H_27_NO_4_ |  |  |  |
| 21 | 8β-acetoxy-11α-hydroxy-lycopodine [22] | 321.194 | C_18_H_27_NO_4_ |  | |  |
| 22 | 8β-hydroxy-11α-acetoxylycopodine [25] | 321.194 | C_18_H_27_NO_4_ |  | |  |
| 23 | lycofawcine [25] | 323.2097 | C_18_H_29_NO_4_ |  | |  |
| 24 | acetylfawcettiine [22] | 349.2253 | C_20_H_31_NO_4_ |  | |  |
| 25 | 12β-hydroxyacetylfawcettiine [22] | 365.2202 | C_20_H_31_NO_5_ |  | |  |
| 26 | 11α-hydroxy-acetylfawcettiine [25] | 365.2202 | C_20_H_31_NO_5_ |  | |  |
| 27 | acetyllycofawcine [25] | 365.2202 | C_20_H_31_NO_5_ |  | |  |
| **alkaloid of lycodine type** | | | | | |  |
| 28 | Lycodine [25] | 258.1732 | C_16_H_22_N_2_O |  | |  |
| 29 | des-N-methyl-α-obscurine [22] | 260.1889 | C_16_H_24_N_2_O |  | |  |
| 30 | α-obscurine [20] | 274.2045 | C_17_H_26_N_2_O |  | |  |
| **alkaloid of fawcettidine type** | | | | | |  |
| 31 | lycopoclavamine A [22] | 261.1729 | C_16_H_23_NO_2_ |  | |  |
| 32 | 8-deoxy-13dehydroserratinine | 261.1729 | C_16_H_23_NO_2_ |  | |  |
| 33 | fawcettimine [21] | 263.1885 | C_16_H_25_NO_2_ |  | |  |
| 34 | obscurinene [22] | 272.1889 | C_17_H_24_N_2_O |  | |  |
| 35 | 14,15-dehydrolycoflexine | 273.1729 | C_17_H_23_NO_2_ |  | |  |
| 36 | lycojaponicumin C [24] | 273.1729 | C_17_H_23_NO_2_ |  | |  |
| 37 | 6-hydroxyl-6,7-dehydro-8-deoxy-13-dehydroserratinine [24] | 275.1521 | C_16_H_21_NO_3_ |  | |  |
| 38 | lycoflexine [21] | 275.1885 | C_17_H_25_NO_2_ |  | |  |
| 39 | lycojapodine A [21] | 277.1678 | C_16_H_23_NO_3_ |  | |  |
| 40 | palhinine B [24] | 277.1678 | C_16_H_23_NO_3_ |  | |  |
| 41 | (15R)-14,15-dihydroepilobscurinol [24] | 277.2042 | C_17_H_27_NO_2_ |  | |  |
| 42 | 6-hydroxyl-6,7-dehydrolycoflexine [24] | 289.1678 | C_17_H_23_NO_3_ |  | |  |
| 43 | lycojaponicumin A [7] | 291.1471 | C_16_H_21_NO_4_ |  | |  |
| 44 | palhinine A [24] | 291.1834 | C_17_H_25_NO_3_ |  | |  |
| 45 | lycojaponicumin B [7] | 293.1627 | C_16_H_23_NO_4_ |  | |  |
| **alkaloid of misceijianeous type** | | | | | |  |
| 46 | phlegmarine | 264.2565 | C_17_H_32_N_2_ |  | |  |
| **triterpenoid** | | | | | |  |
| 47 | Stigmasterol | 400.3705 | C_28_H_48_O |  | |  |
| 48 | β-sitosterol | 412.3705 | C_29_H_48_O |  | |  |
| 49 | serrat-14-ene-3β,21β-diol [26] | 442.3811 | C_30_H_50_O_2_ |  | |  |
| 50 | serrat-14-ene-3β,21α-diol [26] | 442.3811 | C_30_H_50_O_2_ |  | |  |
| 51 | 26-nor-8-oxo-α-onocerin [27] | 444.3603 | C_29_H_48_O_3_ |  | |  |
| 52 | lycojaponicuminol B [27] | 444.3603 | C_29_H_48_O_3_ |  | |  |
| 53 | lycojaponicuminol A [27] | 456.3603 | C_30_H_48_O_3_ |  | |  |
| 54 | lycoclavanol [9] | 458.376 | C_30_H_50_O_3_ |  | |  |
| 55 | 3-epilycoclavanol [9] | 458.376 | C_30_H_50_O_3_ |  | |  |
| 56 | 3β,21β,24-trihydroxyserrat-14-ene [27] | 458.376 | C_30_H_50_O_3_ |  | |  |
| 57 | 3α,21β,24-trihydroxyserrat-14-ene [27] | 458.376 | C_30_H_50_O_3_ |  | |  |
| 58 | α-onocerin [9] | 458.4124 | C_31_H_54_O_2_ |  | |  |
| 59 | lycojaponicuminol C [27] | 460.3553 | C_29_H_48_O_4_ |  | |  |
| 60 | (3β,8β,14α,21α)-26,27-dinoronocerane-3,8,14,21-tetrol [9] | 464.3866 | C_29_H_52_O_4_ |  | |  |
| 61 | (3β,8β,14α,21β)-26,27-dinoronocerane-3,8,14,21-tetrol [9] | 464.3866 | C_29_H_52_O_4_ |  | |  |
| 62 | 3R,21α,24-trihydroxyserrat-14-en-16-one [26] | 472.3553 | C_30_H_48_O_4_ |  | |  |
| 63 | phlegmaric acid [27] | 472.3553 | C_30_H_48_O_4_ |  | |  |
| 64 | lycernuic acid A [27] | 472.3553 | C_30_H_48_O_4_ |  | |  |
| 65 | lycoclaninol [9] | 474.3709 | C_30_H_50_O_4_ |  | |  |
| 66 | japonicumin A [8] | 474.3709 | C_30_H_50_O_4_ |  | |  |
| 67 | 3,20β,21β,24-tetrahydroxyserrat-14-ene [27] | 474.3709 | C_30_H_50_O_4_ |  | |  |
| 68 | 16-oxo-3α-hydroxyserrat-14-en-21α-ol [27] | 484.3916 | C_32_H_52_O_3_ |  | |  |
| 69 | japonicumin B [8] | 488.3502 | C_30_H_48_O_5_ |  | |  |
| 70 | lycopodiin A [9] | 488.3866 | C_31_H_52_O_4_ |  | |  |
| 71 | japonicumin C [8] | 492.3815 | C_30_H_52_O_5_ |  | |  |
| 72 | lycojaponicuminol F [27] | 514.4022 | C_33_H_54_O_4_ |  | |  |
| 73 | β-carotenoid | 576.439 | C_35_H_60_O_6_ |  | |  |
| 74 | lycojaponicuminol D [27] | 604.4128 | C_39_H_56_O_5_ |  | |  |
| 75 | lycojaponicuminol E [27] | 604.4128 | C_39_H_56_O_5_ |  | |  |
| **volatile constituent** | | | | | |  |
| 76 | thymol | 150.1045 | C_10_H_14_O |  | |  |
| 77 | decano ic acid | 172.1463 | C_10_H_20_O_2_ |  | |  |
| 78 | benzene,1,2-dimethoxy-4-propyl- | 180.115 | C_11_H_16_O_2_ |  | |  |
| 79 | α-lonone | 192.1514 | C_13_H_20_O |  | |  |
| 80 | α-Guaiene | 204.1878 | C_15_H_24_ |  | |  |
| 81 | α-cedrol | 222.1984 | C_15_H_26_O |  | |  |
| 82 | pentadecanoic acid | 242.2246 | C_15_H_30_O_2_ |  | |  |
| 83 | palmitic acid | 256.2402 | C_16_H_32_O_2_ |  | |  |
| 84 | farnesyl acetone | 262.2297 | C_18_H_30_O |  | |  |
| 85 | 6,10,14-trimethyl-2-Pentadecanone | 268.2766 | C_18_H_36_O |  | |  |
| 86 | nonadecane | 268.313 | C_19_H_40_ |  | |  |
| 87 | dibutyl phthalate | 278.1518 | C_16_H_22_O_4_ |  | |  |
| 88 | N eophy tadiene | 278.2974 | C_20_H_38_ |  | |  |
| 89 | 9, 12-octadecadieno ic acid | 280.2402 | C_18_H_32_O_2_ |  | |  |
| 90 | cis-11-octadecenoic acid | 282.2559 | C_18_H_34_O_2_ |  | |  |
| 91 | N-eicosane | 282.3287 | C_20_H_42_ |  | |  |
| 92 | stearic acid | 284.2715 | C_18_H_36_O_2_ |  | |  |
| 93 | heptadecane, 2, 6, 10, 15-tetramethyl | 296.3443 | C_21_H_44_ |  | |  |
| 94 | n-Docosane | 310.36 | C_22_H_46_ |  | |  |
| 95 | tricosane | 324.3756 | C_23_H_48_ |  | |  |
| 96 | tetracosane | 338.3913 | C_24_H_50_ |  | |  |
| 97 | pentacosane | 352.4069 | C_25_H_52_ |  | |  |
| 98 | hexacosane | 366.4226 | C_26_H_54_ |  | |  |
| 99 | heptacosane | 380.4382 | C_27_H_56_ |  | |  |
| 100 | octacosane | 394.4539 | C_28_H_58_ |  | |  |
| 101 | nonacosane | 408.4695 | C_29_H_60_ |  | |  |
| 102 | triacosane | 422.4852 | C_30_H_62_ |  | |  |
| 103 | hentriacontane | 436.5008 | C_31_H_64_ |  | |  |
| 104 | dotriacontane | 450.5165 | C_32_H_66_ |  | |  |
| **others** | | | | | |  |
| 105 | ferulic acid | 194.0579 | C_10_H_10_O_4_ |  | |  |
| 106 | japonicumin D [8] | 228.1725 | C_13_H_24_O_3_ |  | |  |
| 107 | clavatine | 263.1885 | C_16_H_25_NO_2_ |  | |  |
| 108 | 6-methyl ether- emodin | 284.0685 | C_16_H_12_O_5_ |  | |  |
| 109 | fawcettiine | 307.2147 | C_18_H_29_NO_3_ |  | |  |
| 110 | di-2-ethylhexyl phthalate | 390.277 | C_24_H_38_O_4_ |  | |  |
| 111 | N-octadecyl alcohol | 410.4488 | C_28_H_58_O |  | |  |
| 112 | betulin | 442.3811 | C_30_H_50_O_2_ |  | |  |
| 113 | clavatol | 450.3709 | C_28_H_50_O_4_ |  | |  |
| 114 | 3β,21β-Dihydroxyserrat-14-en-16-one | 456.3603 | C_30_H_48_O_3_ |  | |  |
| 115 | 16-oxodiepiserratenediol | 456.3603 | C_30_H_48_O_3_ |  | |  |
| 116 | 16-oxoserratenediol | 456.3603 | C_30_H_48_O_3_ |  | |  |
| 117 | 16-oxolycoclavanol | 472.3553 | C_30_H_48_O_4_ |  | |  |
| 118 | lycoclavanin | 488.3502 | C_30_H_48_O_5_ |  | |  |
| 119 | lyclaninol | 488.3866 | C_31_H_52_O_4_ |  | |  |
| 120 | 16-oxolyclanitin | 504.3451 | C_30_H_48_O_6_ |  | |  |
| 121 | 16-oxolyclanitin 30-4-hydroxycinnamoyl | 650.3819 | C_39_H_54_O_8_ |  | |  |
